# Reasons Influencing Long-Term Anticoagulant Treatment Beyond 6 Months for Cancer-Associated Thrombosis in USCAT, A 432-Patient Retrospective Non-Interventional Study

**DOI:** 10.26502/jcsct.5079122

**Published:** 2021-08-11

**Authors:** Ludovic Plaisance, Céline Chapelle, Silvy Laporte, Benjamin Planquette, Laurent Bertoletti, Nicolas Falvo, Francis Couturaud, Lionel Falchero, Isild Mahé, Hélène Helfer, Sadji Dennaoui, Guy Meyer, Isabelle Mahé

**Affiliations:** 1 Hôpital Louis Mourier, APHP, Colombes, Université de Paris, Innovative Therapies in Haemostasis, INSERM, F-75006 Paris, France; 2 Jean Monnet University, University of Lyon, Clinical Research, Innovation, Pharmacology Unit, Saint-Etienne, France; 3 Georges Pompidou European Hospital, University of Paris, Innovative Therapies in Haemostasis, Paris, France; 4 Service of Vascular and Therapeutic Medicine, Vascular Dysfunction and Hemostasis Team, Jean-Monnet University, Saint-Etienne, France; 5 Department of Internal Medicine, Dijon University Hospital, Dijon, France; 6 Hôpital de la Cavale Blanche CHRU de Brest, University Hospital of Brest, Brest, France; 7 North-West Hospital Villefranche-sur-Saône, Gleize, France; 8 F-CRIN INNOVTE network, Saint-Etienne, France

**Keywords:** Cancer, Thrombosis, Anticoagulants, Treatment Duration

## Abstract

**Background and objectives:**

Few data are available about anticoagulation management beyond 6 months in patients with cancer associated thrombosis (CAT). Our objective was to describe anticoagulant treatment modalities up to 12 months.

**Methods:**

The management of the anticoagulant treatment beyond 6 months was described in this initially retrospective non-interventional French multicenter study in patients treated with low-molecular-weight heparins (LMWH) still alive at the end of an initial 6-month treatment period. Clinical outcomes, including venous thromboembolism, recurrence, bleeding and deaths have been published previously.

**Results:**

Among the 432 patients (mean age 66.5±12.7 years) included in the study, 332 were followed up to 12 months while 96 patients deceased before study end and 4 patients were lost-to-follow-up. At 6 months, anticoagulant therapy was stopped in 74 patients, 56 were switched to vitamin K antagonists (VKA) (16.1% [95%CI, 12.4%–20.4]), 30 to direct oral anticoagulants (DOAC) (8.6% [95%CI, 5.9%–12.1]). LMWHs were maintained in 256 patients (73.6% [95%CI, 68.6–78.1]). During the follow-up, LMWHs were definitively discontinued in 86 patients (33.7%), the main reason being a favorable course of the cancer (16 patients, 18.6%), or the thromboembolic disease (11 patients, 12.8%), whereas concern about bleeding risk was low (2 patients, 2.3%).

**Conclusion:**

Anticoagulation beyond 6 months and up to 12 months was in accordance with clinical practice guidelines suggesting that treatment should be continued as long cancer is active or in the absence of bleeding risk. Anticoagulant treatment discontinuation beyond 6 months was influenced by the favorable courses of both malignancy and thromboembolic disease, as well as patient’s preference.

## 1. Introduction

The risk of venous thromboembolism (VTE) in patients with cancer is 7-fold higher compared to patients without cancer [1]. Managing patients with CAT represents a significant challenge since they are at higher risk of both VTE recurrence and major bleeding compared to patients without cancer [2, 3]. Clinical practice guidelines for the treatment of CAT recommend a minimum of 6 months anticoagulant treatment duration [4–8]. The rate of adherence to guidelines including the use of low-molecular-weight heparins (LMWH) remains poorly documented. In the USA, before the introduction of direct oral anticoagulants (DOAC), warfarin and LMWH were used in 50% and 40% of patients, respectively. Over 6 months, only 13% of patients who initiated LMWH remained on them while 30% remained on oral anticoagulants. Also, more patients switched from LMWH to warfarin and other anticoagulants (44%) versus those who switched from warfarin (28%) [9]. In a cohort of 372 European CAT patients treated with LMWH followed up to 6 months the cumulative incidence of discontinuation was 21% after a median period of 90 days and one of five patients stopped LMWH injections because of side effects [10]. There is no established consensus on the optimal duration of the anticoagulant treatment in patients with CAT, especially beyond 6 months. Most guidelines tend to recommend the extension of the anticoagulant therapy for as long as the cancer is active and/or the patient receives an antineoplastic treatment which may be associated with an increased risk of VTE recurrence although these recommendations are not based on randomized trials [4–8].

Less is known about reasons influencing anticoagulant treatment strategy beyond 6 month in real-world practice. Several studies mostly uncontrolled have evaluated extended anticoagulant therapy for patients with CAT suggesting that long-term anticoagulant treatment beyond 6 months may be associated with a lower risk of VTE recurrence and bleeding compared to the initial 6-month treatment period [5, 11–14]. However, studies available to date provide limited orientation for the management of CAT patients beyond 6 months after the index VTE mainly due to the absence of randomization, the relatively small patient sample size, and populations not comparable across studies (Table 1). Two large prospective cohort studies have documented the treatment of CAT patients initially treated with tinzaparin for 6 months [15] (https://clinicaltrials.gov/ct2/show/NCT02898051). We previously published the results of the USCAT study, a retrospective non-interventional study with the objective to briefly describe in the real-world practice the use of the anticoagulant treatment beyond 6 months and up to 12 months following index VTE and to document clinical outcomes i.e. VTE recurrence, bleeding and deaths in CAT patients initially treated for 6 months in both PREDICARE and aXa studies [16]. In this analysis, we focused on reasons influencing the management on the anticoagulant treatment beyond 6 months in the USCAT patient population and particularly reasons for either discontinuing LMWHs or switching them to other anticoagulants.

## 2. Material and Methods

### 2.1 Study design and population

The design and the main results of the USCAT study have been previously described [16]. Briefly, adult patients with cancer and objectively diagnosed acute VTE previously included in both prospective observational cohort studies aXa and PREDICARE and who were still alive and having given their consent for the use of their data were eligible. All 432 participants were enrolled in 59 French hospital centers from August 4, 2011 and April 21, 2016 [16]. The USCAT study was approved by the Institutional Review Board of the University Hospital of Saint-Etienne, France (Institutional Review Board: IORG0007394, Number RBN342018/CHUSTE).

### 2.2 Study objective

Main study objective was to describe the anticoagulant treatment for the management of CAT patients from the 6th month to the 12th month following the index VTE.

### 2.3 Data management and statistics

The same independent investigator had access to the primary data and reviewed medical records of all included patients and recorded data in a standardized case report form. Relevant data, including patient demographics, cancer status and treatment, and anticoagulant therapy were collected up to 12 months after the index VTE event. Information on changes in cancer status, cancer treatment and anticoagulant therapy, specifically notified reasons influencing changes in strategy (as favorable cancer or VTE course, major bleeding, patient’s preference or physician’s decision) was collected. Continuous data were expressed as mean ± standard deviation or median and categorical variables were expressed as absolute numbers (percentages).

The rate of patients continued on LMWHs, VKAs or DOACs beyond 6 months and their 95% confidence interval (95% CI) were also presented. The duration of treatment before the definitive discontinuation of anticoagulation or the switch to another anticoagulant depending on the reasons for definitive discontinuation or switch were graphically summarized by boxplots. Statistical analyses were performed using SAS version 9.4 software (SAS Institute Inc, Cary, NC, USA) and graphics were performed using R statistical software, version 3.6.2.

## 3. Results

Between 4 August 2011 and 21 April 2016, 719 patients were included in aXa and PREDICARE studies in 59 French centers, of which 432 participated in the long-term follow-up of USCAT study [16].

### 3.1 Anticoagulant treatment strategy beyond 6 months post-index event

Therapeutic strategy at 6 months is available for 422 patients of the 432 included patients. Patient’s characteristics according to the anticoagulant treatment strategy at the end of the initial 6-month treatment is summarized in table 2. At 6 months the anticoagulant treatment was continued in 348 patients (82.5%) while it was discontinued in 74 patients (17.5%) of whom 60 patients before the inclusion and 14 patients within the first month of the inclusion in the USCAT study.

### 3.2 Influence of tumor status

The anticoagulant treatment beyond 6 months was mostly continued in pancreas (100% continued vs none discontinued) and upper gastrointestinal cancers (92.3% continued vs 7.7% discontinued). Among the 311 patients with a cancer stage of 3–4, treatment beyond 6 months, was continued in 277 patients (89.1%) while the anticoagulant treatment was discontinued in 34 patients (10.9%). Similar observations were made among 211 patients with cancer in progression as 187 patients (88.6%) had the treatment continued beyond 6 months while 24 patients (11.4%) had the treatment discontinued at 6 months. Conversely, among the 66 patients with tumor remission, treatment had been discontinued in 22 patients (33.3%) and continued in 44 patients (66.7%).

### 3.3 Choice of the anticoagulant

**Figure 1:**
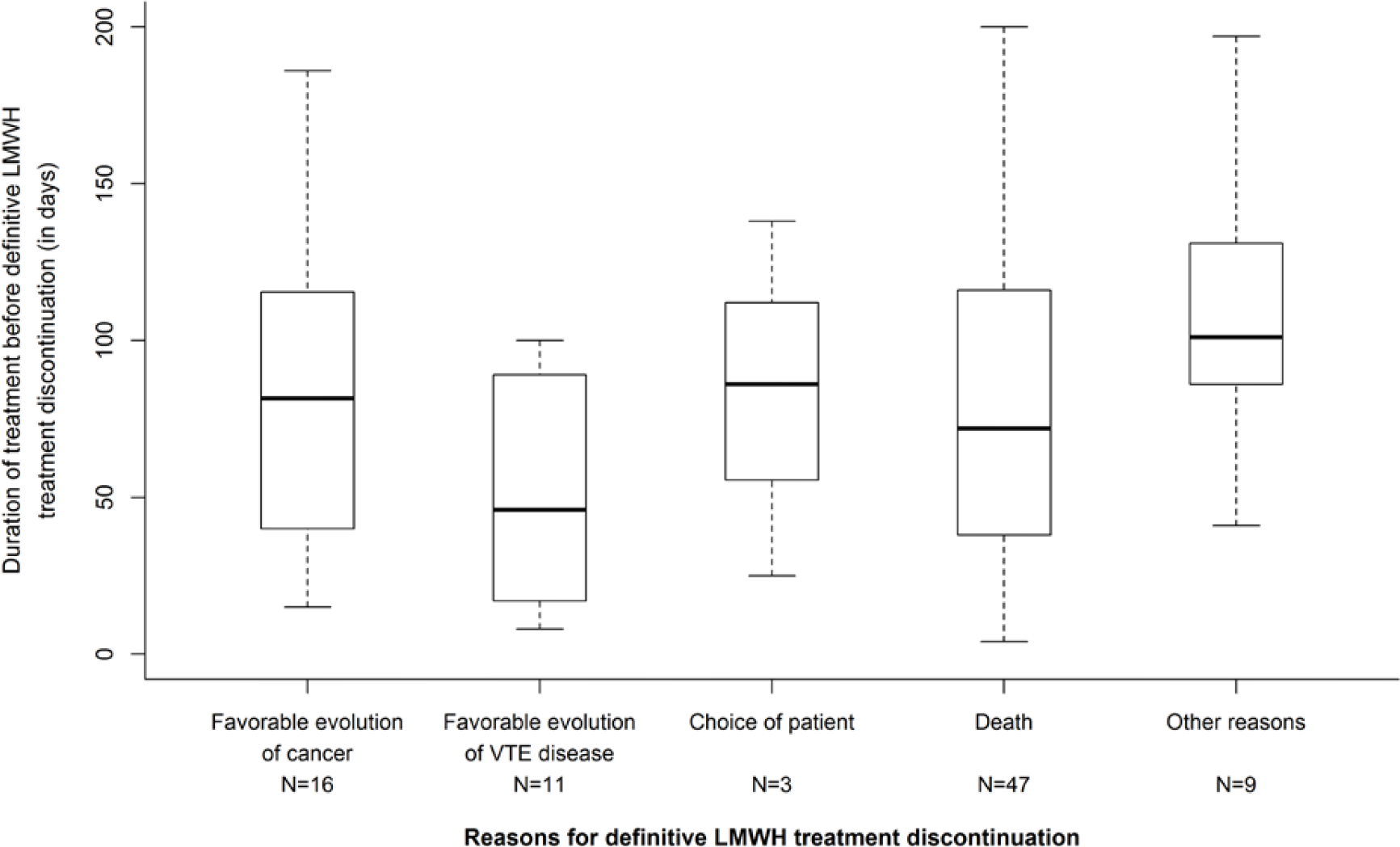
Time to- and main reasons for LMWH permanent discontinuation beyond 6 months after the initial VTE.

**Figure 2:**
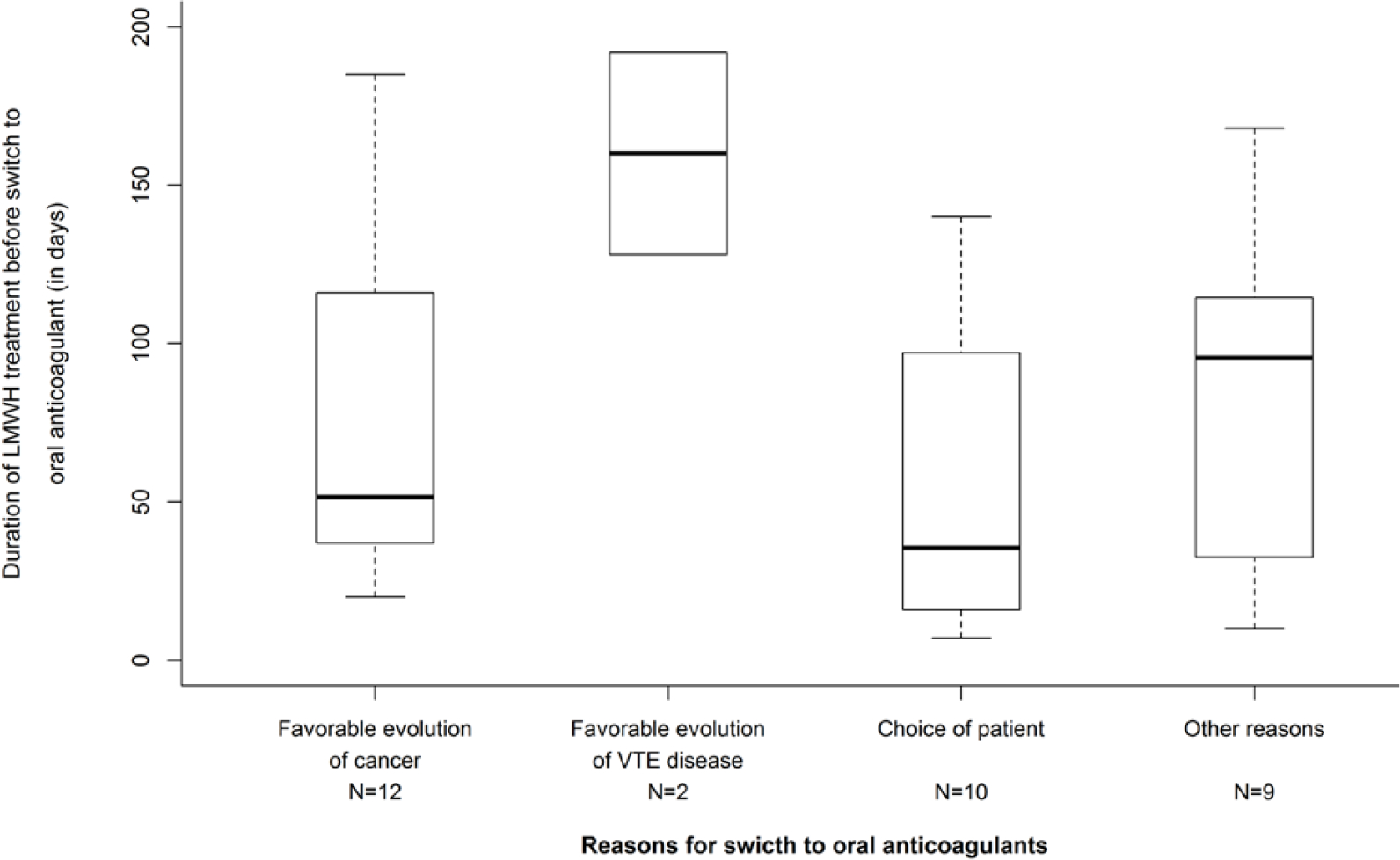
Time to- and main reasons for switching from LMWH to oral anticoagulants (DOAC or VKA) beyond 6 months after the initial VTE.

Beyond 6 months, anticoagulant treatment was continued in 348 patients and main anticoagulants used were LMWHs, VKAs and DOACs (Table 3). Among the 348 patients in whom the anticoagulant treatment was continued beyond 6 months, LMWHs were given in 256 patients (73.6%) for a median treatment duration of 137 days resulting in a median total treatment duration since the index event of 292 days. Most of patients were given tinzaparin (n=245/348) for similar median treatment durations. From the 6^th^ to the 12^th^ month, 86 patients (33.7%) treated with LMWH had their treatment definitively discontinued mainly due to the favorable evolution of the cancer disease (n=16) or the favorable course of the thromboembolic disease (n=11) after a median treatment duration beyond 6 months of 81.5 and 46.0 days, respectively. Only 3 definitive LMWH discontinuations were related to patient’s decision (Figure 1). A temporary discontinuation of LMWHs was reported in 16 patients (6.3%). Switch to another anticoagulant was reported in 42 patients, mainly to oral VKAs (n=20) and DOACs (n=13) (Table 3), guided by the favorable evolution of the cancer disease (n=12) and choice of the patient (n=10) (Figure 2) after a median treatment duration of 51.5 and 35.5 days, respectively. Changes in the LMWHs doses were reported in 30 patients (11.8%) with a dose increase in 10 patients and a dose decrease in 20 patients (Table 3).

A total of 56 patients (16.1%) remained on VKAs beyond 6 months for a median treatment duration of 183 days. VKAs were definitively discontinued in 12 patients (21.8%) for reasons unrelated to safety concerns while the treatment was temporarily discontinued in 6 patients (10.9%). A switch of VKAs was reported in 8 patients (14.5%) mainly to LMWHs. DOACs were continued in 30 patients (8.6%) beyond the initial 6-month treatment period for a median treatment duration of 183 days. DOACs were definitively discontinued in 8 patients (27.6%) for other reasons than safety concern. There were no temporary discontinuation or switch to another treatment. Unfractionated heparin (UFH) and fondaparinux were each used beyond 6 months in 3 patients (0.9%).

## 4. Discussion

The description of the anticoagulant treatment in USCAT represents an additional information on the management of CAT patients up to 12 months after the index event in the real world of clinical practice. As previously published [16], a total of 432 patients initially treated with the LMWH tinzaparin and completing a 6-month follow-up were included in the USCAT study, of whom 332 were still alive and followed at m12 with a median treatment duration beyond 6 months of 137, 183 and 183 days with LMWHs, VKAs and DOACs, respectively which is in accordance with results from studies summarized in Table 1.

### 4.1 Treatment strategy beyond 6 months

At inclusion in USCAT study, i.e. 6 months after the VTE index event, 348/422 patients (82.5%) had an anticoagulant treatment. Anticogulant treatment was mainly continued in patients with stage 3–4 (89.1%) and in patients with cancer progression (88.6%). This is consistent with guidelines which recommend the continuation of the anticoagulant treatment beyond 6 month after the VTE index event when cancer is considered as active [4–8]. In our analysis, the assessment of the anticoagulant treatment strategy beyond 6 months was approximatively made at the end of the initial 6-month period regarding either the discontinuation (Figure 1) or the extension of the anticoagulant, or the switch to another anticoagulant. Based on observations in previous studies, the therapeutic strategy was based on clinical criteria such as patient’s condition and preference, course of malignancy and course of thromboembolic disease [14, 17]. This substantially differs from the approach during the first 6 months in which increased bleeding risk, toterability, acceptability, and poor prognosis are key factors that influence discontinuing the anticoagulant treatment or to switching to another anticoagulant.

### 4.2 Anticoagulant treatment usefulness beyond 6 months

Regarding the 7-to-12-month VTE recurrence raw incidence, previous observational studies have emphasized the usefulness of extended anticoagulant treatment beyond 6 months [11–13] whereas international guidelines leave the maintenance of the anticoagulant treatment and the choice of the anticoagulant to the physician’s judgment on a case-by-case based on the expected benefit-risk balance [4–8]. In the USCAT study, of the 348 patients in whom the anticoagulant treatment was continued beyond 6 months, a majority (n=256) received a LMWHs, mostly tinzaparin (n=245), while 56 and 30 patients received VKAs and DOACs, respectively.

### 4.3 Reasons incluencing the anticoagulant treatment strategy beyond 6 months

During the 6-to-12-month follow-up the treatment was definitively discontinued in 86 (33.7%), 12 (21.8%) and 8 patients (27.6%) treated with LMWHs, VKAs and DOACs, respectively. The main reason for definitive LWMH discontinuation was based on physician’s judgment, when the overall patient condition was considered at lower risk of VTE recurrence related to a favorable course of the cancer or of the thromboembolic disease (Figure 1). Little concern was reported about bleeding risk regarding treatment discontinuation. In the HOKUSAI VTE-Cancer extension study, investigator’s decision based on the estimated benefit-risk, patient’s preference relative to the inconvenience of dosing, and cancer considered as cured, were emphasized as main reasons for permanent discontinuation of dalteparin therapy beyond 6 months [11]. The decisions based on physician’s judgment are in accordance with clinical practice guidelines suggesting that treatment should be continued as long the cancer is active or in the absence of bleeding risk and consistent with previous observations in France emphasizing a case-by-case approach for the management of the anticoagulant treatment in CAT patients [4, 16, 18, 19]. Our observations are consistent with data on the acceptability of LMWH treatment in CAT patients, as the TROPIQUE qualitative study showed high rates of convenience and treatment satisfaction after 6-month treatment with LMWH [20]. DOACs may represent an alternative for convenience and treatment satisfaction as in COSIMO trial [21].

### 4.4 Reasons influencing LMWH switch to an oral anticoagulant

Among 256 patients in whom LMWH was continued beyond -6 months, treatment was switched to another anticoagulant treatment in 42 patients, mostly to VKAs and DOACs in 20 (47,6%) and 13 (31.0%) patients, respect-tively. The decision to switch to an oral anticoagulant, usually made around the 6^th^ month, was based of patient’s preference carefully assessed by the physician (Figure 2). Our results are consistent with FRONTLINE 2 [22] in which interviewed physicians were maintaining the anticoagulation after initial heparin, by using oral anticoagulant medications in the longer term. In USCAT, the oral anticoagulation beyond 6 months mainly consisted of VKAs in 56 patients. In these patients most anticoagulant switches were made to LMWHs. The main reason may have been that at the time of the initiation of aXa and PREDICARE studies, DOACs were not usually recommended for the treatment of CAT patients. Previous publications have considered DOACs as an alternative to LMWH for convenience and treatment satisfaction reasons as shown in the COSIMO trial [21]. This non intervenetionnal study aimed at evaluating patient-reported treatment satisfaction following a switch from standard of care (SOC) (more than 4 weeks of LMWH or VKA therapy) to rivaroxaban for the treatment of CAT. It suggested an increase in treatment satisfaction in CAT patients when switching to rivaroxaban due to better convenience and acceptability than SOC therapy (mostly LMWH).

Furthermore, a discrete choice experiment on COSIMO study patients preferences revealed that the main reason guiding the patient willingness to switch to oral anticoagulation was that oral route of administration was associated with better convenience [21].

### 4.5 Study limitations

Given the observational retrospective nature of our study, limitations were related to the absence of randomization and several missing data that made difficult the comparison of different treatment strategies. Another potential limitation is that DOAC’s were considered as emerging therapeutic options at the time of PREDICARE and aXa studies initiation when predominantely VKA and LMWH were available for the treatment of CAT.

### 4.6 Study strengths

USCAT is the largest observational study to date on CAT patients treated with LMWH beyond 6 months after the VTE index event (N = 432) while a large proportion of patients have completed 12-month follow-up (n=332). Clinical protocols and event adjudication in PREDICARE [15] and aXa (NCT02898051; https://clinicaltrials.gov/ct2/show/NCT02898051) studies were homogeneous, allowing the inclusion of a large sample size in USCAT. Moreover, patients characteristics were documented 6 months after the index VTE i.e., at the time when the treatment strategy for the subsequent 6 months was to be discussed. Reasons influencing therapeutic decisions for the extended anticoagulant treatment with LMWHs beyond 6 months in patients with CAT discussed in our study reflect the real-life physician’s experience in the clinical practice.

## 5. Conclusion

In this real-world practice analysis, anticoagulant treatment with LMWH was continued beyond 6 months and up to 12 months in most patients with CAT. However different factors have influenced physician’s decisions beyond the initial 6 months of therapy. Beyond 6 months, treatment discontinuations were mainly related to the favorable course of the malignancy or the thromboembolic disease, while switches to oral anticoagulants were guided by patient’s preference.

Unlike the risk management approach during the initial 6 months, the risk of bleeding was not a major concern that would justify treatment discontinuation beyond 6 months. Based on our observations it therefore seems fundamental to re-assess the anticoagulation strategy after the initial 6 months of therapy to optimize physician’s decision. Establishing more formal clinical practice guidelines for the long-term anticoagulant treatment in CAT patients beyond 6 months with the identification of therapeutic decision making factors may require further clinical research in the form of prospective controlled trials.

## Figures and Tables

**Table 1:** Anticoagulant treatment duration beyond 6 months in previous studies.

	Daltecan [12]	Ticat [13]	Schmidt et al. [14]	Hokusai VTE Cancer Extension [11]		RIETE [23]	
	Dalteparin n=185*	Tinzaparin n=247	n=322	Dalteparin n=525	Edoxaban n=525	LMWH n=482**	VKA n=482**
Mean duration (± SD)	210 days	15.6 ± 13.2 months	NA	NA	NA	323.9 ± 207.1 days	441.6 ± 378.0 days
Median duration (IQR)	NA	NA	NA	318 days (216, 360)	351 days (272, 364)	256 days (209, 368)	309 days (219, 503)
Anticoagulant continuation n (% )	109 (59.8 %)	198 (80.2%)	222 (68.9%)	273* (52%)	294*(56%)	NA	NA
Switch to another anticoagulant n (%)	NA	NA	NA	34 (6%)	19 (4%)	NA	NA
				−to DOAC: 12 (4.4%)	−to DOAC: 4 (1.4% )		
				−to VKA: 1 (0.4%)	−to VKA: 0 (0%)		
				−to LMWH: 21 (7.7%)	−to LMWH: 15 (5.1%)		

SD: standard deviation; IQR: interquartile range

*2^nd^ 6 month cohort

** after propensity score matching beyond 6 month of initial anticoagulation treatment

**Table 2:** Baseline demographic and clinical characteristics of included patients according to therapeutic strategy at 6 months.

	Anticoagulant at 6 months*	
	Continued N = 348	Discontinued N = 74
Mean age (years) ± SD	66.6 ± 12.7	65.7 ± 12.7
Age ≥ 75 years, no. (%) Row percentages	103 (29.6) 83.7	20 (27.0) 16.3
Male sex, no. (%) Row percentages	169 (48.6) 83.3	34 (45.9) 16.7
**Site of cancer disease, no. (%)**		
Solid tumor Row percentages	321 (92.2) 83.4	64 (86.5) 16.6
Colorectal Row percentages	68 (21.2) 80.0	17 (26.6) 20.0
Lung Row percentages	64 (19.9) 84.2	12 (18.8) 15.8
Breast Row percentages	57 (17.8) 86.4	9 (14.1) 13.6
Genitourinary Row percentages	47 (14.6) 79.7	12 (18.8) 20.3
Gynecologic Row percentages	33 (10.3) 80.5	8 (12.5) 19.5
Pancreas Row percentages	13 (4.0) 100.0	0 (0) 0.0
Upper gastrointestinal Row percentages	12 (3.7) 92.3	1 (1.6) 7.7
Hepatobiliary Row percentages	7 (2.2) 77.8	2 (3.1) 22.2
Other Row percentages	20 (6.2) 87.0	3 (4.7) 13.0
Hematologic malignancy Row percentages	21 (6.0) 70.0	9 (12.2) 30.0
Non-Hodgkin lymphoma Row percentages	11 (52.4) 78.6	3 (33.3) 21.4
Multiple myeloma Row percentages	4 (19.0) 57.1	3 (33.3) 42.9
Leukemia Row percentages	5 (23.8) 71.4	2 (22.2) 28.6
Hodgkin lymphoma Row percentages	1 (4.8) 50.0	1 (11.1) 50.0
Other type of tumor Row percentages	6 (1.7) 85.7	1 (1.4) 14.3
**Stage (N = 390), no. (%)**		
1 Row percentages	27 (8.6) 61.4	17 (26.2) 38.6
2 Row percentages	11 (3.5) 44.0	14 (21.5) 56.0
3 or 4 Row percentages	277 (87.9) 89.1	34 (52.3) 10.9
**Cancer evolution (N = 424), no. (%)**		
Remission Row percentages	44 (12.9) 66.7	22 (30.6) 33.3
Stability Row percentages	111 (32.5) 81.0	26 (36.1) 19.0
Progression Row percentages	187 (54.7) 88.6	24 (33.3) 11.4
**Index VTE** ^**$**^ **, no. (%)**		
PE± DVT Row percentages	262 (75.7) 83.7	51 (68.9) 16.3
Proximal DVT (N = 429) Row percentages	92 (26.6) 84.4	17 (23.3) 15.6
Distal DVT (N = 429) Row percentages	85 (24.6) 81.0	20 (27.4) 19.0

The figures in italics correspond to the percentages of patients who continued or discontinued the anticoagulant treatment at 6 months among the total number of patients who presented the characteristic. Upper gastrointestinal tumor: gastrointestinal and oesophagus. Other solid tumor: ENT, cerebral, sarcoma, melanoma and peritoneal. SD: standard deviation; VTE: venous thromboembolism; PE: pulmonary embolism; DVT: deep vein thrombosis; $ more than one event in several patients.

● the information about anticoagulant was available for 422 patients

**Table 3:** Anticoagulant treatments continued in the 348 patients beyond 6 months following the index VTE.

	Anticoagulant continued at 6 months N = 348
LMWH, no. (% [95% CI])	256 (73.6 [68.6–78.1])
Median treatment duration beyond 6 months (days)	137.0
Median total treatment duration since the index VTE (days)	292.0
Permanent discontinuation (N = 255), no. (%)	86 (33.7)
Temporary interruption (N = 255), no. (%)	16 (6.3)
Switch to another anticoagulant (N = 255), no. (%)	42 (16.5)
VKA	20 (47.6)
DOAC	13 (31.0)
Other LMWH	7 (16.7)
UFH	2 (4.8)
Change in the LMWH dosage (N = 255), no. (%)	30 (11.8)
VKA, no. (% [95% CI])	56 (16.1 [12.4–20.4])
Median treatment duration beyond 6 months (days)	183.0
Permanent discontinuation (N = 55), no. (%)	12 (21.8)
Temporary interruption (N = 55), no. (%)	6 (10.9)
Switch to another anticoagulant (N = 55), no. (%)	8 (14.5)
LMWH	7 (87.5)
Fondaparinux	1 (12.5)
Change in the VKA dosage (N = 55), no. (%)	0 (0.0)
DOAC, no. (% [95% CI])	30 (8.6 [5.9–12.1])
Median treatment duration beyond 6 months (days)	183.0
Permanent discontinuation (N = 29), no. (%)	8 (27.6)
Temporary interruption (N = 29), no. (%)	0 (0.0)
Switch to another anticoagulant (N = 29), no. (%)	0 (0.0)
Change in the DOAC dosage (N = 29), no. (%)	0 (0.0)
UFH, no. (% [95% CI])	3 (0.9 [0.2–2.5])
Median treatment duration beyond 6 months (days)	181.5
Permanent discontinuation, no. (%)	1 (33.3)
Temporary interruption, no. (%)	0 (0.0)
Switch to another anticoagulant, no. (%)	0 (0.0)
Change in the UFH dosage, no. (%)	0 (0.0)
Fondaparinux, no. (% [95% CI])	3 (0.9 [0.2–2.5])
Median treatment duration beyond 6 months (days)	183.0
Permanent discontinuation, no. (%)	0 (0.0)
Temporary interruption, no. (%)	0 (0.0)
Switch to another anticoagulant, no. (%)	0 (0.0)
Change in the Fondaparinux dosage, no. (%)	1 (33.3)

SD: standard deviation

VTE: venous thromboembolism

LMWH: low-molecular-weight heparin

VKA: vitamin K antagonist

DOAC: direct oral anticoagulant

UFH: unfractionated heparin

*more than one reason in several patients.

## Appendix: Investigators and Study Centers

Île de France (154 patients, 11 centers)–G. Meyer, Paris, T. Papo, Paris; A. Burnod, Paris, I. Mahé, Colombes; H. Doubre, Suresnes; S. Ropert, Antony; J. Ezenfis, G. Oliviero, Longjumeau I. Monet, Créteil; E. Assaf, Créteil; C. Locher, Meaux; B. Philippe, Pontoise. Auvergne-Rhône-Alpes (94 patients, 9 centers) – L. Bertoletti, Saint-Étienne; J. Schmidt, Clermont-Ferrand; L. Falchero, Villefranche sur Saône; D. Beal-Ardisson, Lyon; D. Pere Verge, Lyon; P. Gérôme, Lyon; V. Granger, Grenoble; P. Cony-Makhoul, Annecy; C. Bettarel-Binon, Montluçon. Bretagne (42 patients, 6 centers) – F. Couturaud, Brest; B. Lucas, Brest; F. Schlurmann-Constans, Quimper; C. Lefevre, Saint-Grégoire; B. Boutruche, Rennes, M. Ferec, Morlaix. Bourgogne-Franche-Comté (41 patients, 3 centers) – N. Falvo, Dijon; F. Ghiringelli, Dijon; C. Faure, Vesoul; Hauts de France (19 patients, 5 centers) – M.A. Sevestre, Amiens; C. Desauw, Lille; A. Scherpereel, Lille; S. Aquilanti, Arras; V. Bourgeois, Boulogne-sur-Mer; Provence-Alpes-Côte d’Azur (35 patients, 8 centers) – A. Elias, Toulon; R. Poyet, Toulon; P. Tomasini, Marseille; P. Debourdeau, Avignon; N. Cloarec, Avignon; J.L. Mouysset, Aix en Provence; T. Benchaa, Aix en Provence; N. Bensahli-Bouhayed, Salon de Provence. Grand Est (16 patients, 7 centers) – B. Mennecier, Strasbourg; D. Stephan, Strasbourg; L.M. Dourthe, Strasbourg; L. Moreau, Colmar; D. Spaeth, Nancy; C. Witte-Seiler, Troyes; N. Jovenin, Saint-Dizier. Nouvelle Aquitaine (9 patients, 2 centers) – J. Constans, Bordeaux; J.C. Saby, Bordeaux. Centre Val de Loire (10 patients, 3 centers) – B. Lemaire, Orléans; J. Meunier, Orléans; C. Lethrosne, S. Vignot, Le Coudray; Pays de la Loire (4 patients, 3 centers) J. Connault, Nantes; D. Cornuault-Foubert, Angers; O. Cojocarascu, Le Mans; Occitanie (4 patients, 1 center) – A. Bura-Rivière, Toulouse. Corse (4 patients,1 center) – A. Frikha, Bastia.
